# Turning off the antennae: nsrC suppresses phycobilisome biosynthesis during nitrogen starvation

**DOI:** 10.1093/plcell/koag309

**Published:** 2026-10-07

**Authors:** Barbara Bourgade

**Affiliations:** Assistant Features Editor, the Plant Cell, American Society of Plant Biologists, United States; Department of Organismal Biology, Uppsala University, 752 36 Uppsala, Sweden

Nitrogen is an essential element for life and is frequently limiting in the environment. Although many cyanobacteria are diazotrophic and can convert atmospheric N_2_ into usable forms, non-diazotrophic cyanobacteria are unable to utilize atmospheric N_2_ and instead rely on dissolved nitrogen sources, such as ammonium, nitrate, and amino acids. In these species, nitrogen limitation reduces growth and photosynthetic activity and triggers extensive metabolic reprogramming (Inabe et al. 2021). One key mechanism for adapting to low nitrogen levels is the targeted degradation of proteins, which releases amino acids that can be recycled for use in critical processes associated with nitrogen limitation responses. Phycobilisomes, the major pigment-protein antenna complexes in cyanobacteria and red algae, are prominent targets of this response. Their structural components, including phycocyanin, allophycocyanin, and linker proteins, are degraded during nitrogen starvation, releasing substantial amounts of amino acids, which reduces their light-harvesting capacity and is manifest as chlorosis or a loss of pigmentation (Spät et al. 2018).

In *Synechococcus* species, the global nitrogen-responsive transcription factor NtcA and the signaling protein PII (Espinosa et al. 2006) contribute to the broad transcriptional and metabolic responses observed as the cells are depleted for nitrogen. During this process, phycobilisome degradation is coordinated by an extensive regulatory network involving several regulatory proteins, including NblA, which promotes the degradation of phycobilisome components such as the phycocyanin β subunit (CpcB) by the ClpP protease (Krauspe et al. 2021). Although the mechanisms controlling phycobilisome degradation during nitrogen starvation have been extensively studied, it remains unclear how their biosynthesis responds to nitrogen starvation.

In recent work, Wenzhe Li and colleagues (Li et al. 2026) identified the small helix-loop-helix Nitrogen Starvation Regulator of the *cpc* operon (NsrC) as a key effector that represses phycobilisome biosynthesis under nitrogen deprivation conditions (Fig. 1). In nitrogen-deplete conditions, *nsrC* transcript levels were 15-fold higher than in nitrogen-replete conditions in the cyanobacterium *Synechoccus elongatus* sp. PCC 7942. This transcriptional upregulation was also associated with an increased abundance of the NsrC protein, consistent with its role in the nitrogen starvation response. To further elucidate the role of NsrC, the authors deleted *nsrC* and the resulting mutant displayed increased photosynthetic O_2_ evolution and phycobiliprotein content, for both phycocyanin and allophycocyanin, under nitrogen-deplete conditions, with no significant difference under nitrogen replete conditions. Furthermore, the complemented strain overexpressing NsrC exhibited a phenotype similar to that of wild-type cells. These findings support a specific role for NsrC during nitrogen starvation.

**Figure 1:**
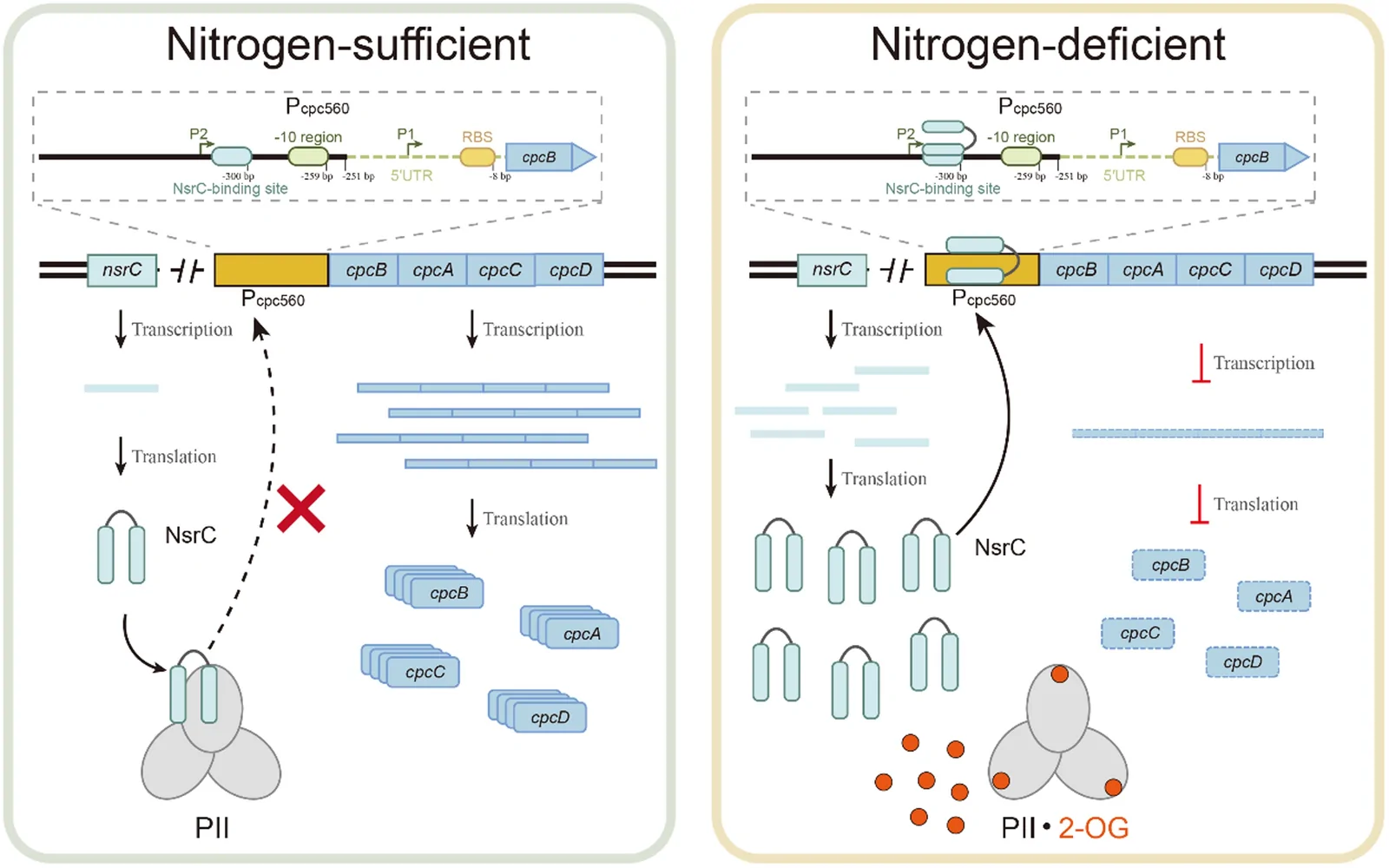
NsrC acts as a nitrogen-dependent regulator of phycobilisome biosynthesis. Under nitrogen-replete conditions (left panel), NsrC interacts with the signalling protein PII, preventing its binding to the promoter P_cpc560_ of the phycocyanin biosynthetic operon. During nitrogen starvation (right panel), increased levels of the metabolite 2-OG weaken the NsrC-PII interaction, enabling NsrC to bind P_cpc560_ and repress transcription of the *cpc* operon, thereby inhibiting phycobilisome biosynthesis. Adapted from Li et al. 2026, Figure 9.

In RNA-sequencing analyses, the *nsrC* mutant displayed upregulation of 115 genes related to photosynthesis and photosynthetic antennae during nitrogen deprivation relative to wild-type cells. Genes encoding phycobilisome components, including the phycocyanin subunits CpcA and CpcB and the allophycocyanin subunits ApcA and ApcB, were markedly upregulated in the mutant, consistent with the elevated phycobilisome content during nitrogen deprivation. To elucidate the role of NsrC in transcriptional regulation, the authors used ChIP-seq and identified a GC-rich DNA motif upstream of the transcriptional start sites of both *cpc* (referred to as promoter P_cpc560_) and *apc* operons with which NsrC interacted. An electrophoretic mobility shift assay validated that NsrC formed a DNA-protein complex with this specific motif of the *cpc* operon (but not with the *apc* operon), demonstrating that NsrC directly binds to the *cpc* regulatory region. Binding affinity measurements showed that NsrC bound to the DNA motif with high affinity and that this interaction was dependent on the motif's specific sequence. Together, these results suggest that NsrC regulates the *cpc* operon and likely plays a direct role repressing transcription when the cells experience nitrogen deprivation.

The authors further demonstrated that NsrC interacted with PII, a signaling protein that senses cellular nitrogen availability through 2-oxoglutarate (2-OG), a metabolite that accumulates during nitrogen starvation. Co-immunoprecipitation experiments detected NsrC–PII interaction under both nitrogen-replete and nitrogen-deplete conditions, but its strength depended on 2-OG concentrations, as demonstrated for other proteins interacting with PII (Krauspe et al. 2021). High 2-OG concentrations weakened the NsrC–PII interaction, whereas low 2-OG concentrations led to strong interaction. Importantly, the interaction with PII affected NsrC binding to the *cpc* regulatory region: strong NsrC–PII interaction under low 2-OG conditions reduced NsrC binding to the *cpc* promoter, whereas elevated 2-OG weakened the interaction and allowed NsrC to bind the promoter. Thus, when the cells are nitrogen starved, accumulation of 2-OG promotes dissociation of the PII–NsrC complex, enabling NsrC to repress *cpc* expression (and perhaps other NsrC-regulated genes), thereby suppressing phycobilisome biosynthesis.

Phycobilisomes are present in cyanobacteria and red algae but are absent in diatoms and other algal lineages. Despite the absence of phycobilisomes in diatoms and brown algae, NsrC homologues can be found in both red and brown algae as well as in diatoms. However, this study suggests functional evolutionary divergence of NsrC homologues in diatoms and brown algae as only *nsrC* from a red alga was able to complement the *nsrC* mutant phenotype in *S. elongatus*. These findings raise questions about how NsrC evolved in association with different light-harvesting systems, the specific genes that it regulates, and how nitrogen-responsive regulation of photosynthetic antennae has diversified across photosynthetic organisms.

In conclusion, Li et al. identified the small helix-loop-helix protein NsrC as a key regulator of phycobilisome biosynthesis under nitrogen starvation. By coupling 2-OG–dependent PII signaling to transcriptional repression of the *cpc* operon, NsrC provides a mechanism to suppress the production of new phycobilisomes during nitrogen starvation, complementing the established degradation of existing antenna complexes through the functions of NblA and ClpP proteases. These findings provide a more complete picture of how cyanobacteria remodel their photosynthetic apparatus in response to nitrogen availability and identify the NsrC regulatory pathway as a potential target for manipulating phycobilisome abundance and light capture in cyanobacteria.

## Recent related articles in *The Plant Cell*:

- Espinoza-Corral et al. (2025) identified ApcI as a red light–induced phycobilisome linker protein that modulates energy transfer between phycobilisomes and PSII, contributing to the acclimation of *Synechocystis* sp. PCC 6803 to red light.
- Zhao et al. (2023) resolved the structure of the PSI–light-harvesting complex I supercomplex in the cryptophyte *Chroomonas placoidea*, revealing how alloxanthin Chl a/*c*-binding proteins extend the spectrum of captured light and providing insights into the evolution of red-lineage photosynthesis.
- Xie et al. (2024) demonstrated that phosphoketolase and KDPG aldolase contribute to the metabolic flexibility of *Synechococcus elongatus* PCC 7942, supporting carbon metabolism during darkness and subsequent recovery of photosynthetic activity.

## Data Availability

No new data was generated for this article.
